# Examining critical assumptions in global conservation practice

**DOI:** 10.1111/cobi.70102

**Published:** 2025-07-03

**Authors:** Kendra Opatovsky, Brian Pentz, Philip A. Loring

**Affiliations:** ^1^ The Agility Lab The Nature Conservancy Arlington Virginia USA; ^2^ Global Science Team The Nature Conservancy Arlington Virginia USA

**Keywords:** assumptions, biodiversity, eNGOs, multiloop learning, theory of change, aprendizaje, biodiversidad, multibucle, ONGe, supuestos, teoría del cambio

## Abstract

The ability of conservation practitioners to design and implement successful conservation projects and scale up positive outcomes depends in large part on their ability to make accurate assumptions about the social and ecological contexts in which their projects operate. To understand the types of assumptions characterizing conservation practice and strategy, we examined 300 assumptions identified by conservation practitioners in project workshops at The Nature Conservancy, a large environmental nongovernmental organization, as being critical to the success of their conservation projects. We identified 7 conservation assumption themes (public attitudes and perceptions; capacity and resources; economic and behavioral factors; government, politics, and policy; impacts and scalability; knowledge and evidence; and organizational or internal factors), which primarily relate to the human dimensions of conservation (e.g., values, human behavior, relationships, policy, and politics). The conservation assumptions focused predominantly on project‐level and place‐based matters, rather than on the root causes of conservation problems. For people‐ and equity‐centered conservation approaches, our findings suggest that conservation teams should systematically engage with areas of elevated uncertainty and should especially focus on axiomatic assumptions made about the broader contexts in which conservation projects operate. These insights can inform effective project design and adaptive learning and can directly improve project success.

## Graphical abstract

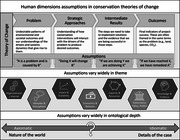

## Abstract (es)

Análisis de los supuestos críticos en la práctica de la conservación global

**Resumen:**

La capacidad de los profesionales de la conservación para diseñar y ejecutar con éxito proyectos de conservación y ampliar los resultados positivos depende en gran medida de su capacidad para hacer suposiciones precisas sobre los contextos sociales y ecológicos en los que operan sus proyectos. Para comprender los tipos de supuestos que caracterizan la práctica y la estrategia de la conservación, examinamos 300 supuestos identificados por profesionales de la conservación en los talleres de proyectos de The Nature Conservancy, una gran organización no gubernamental medioambiental, como fundamentales para el éxito de sus proyectos de conservación. Identificamos siete temas de supuestos de conservación (actitudes y percepciones del público; capacidad y recursos; factores económicos y de comportamiento; gobierno, política y política; impactos y escalabilidad; conocimiento y pruebas; y factores organizativos o internos), que se relacionan principalmente con las dimensiones humanas de la conservación (por ejemplo, valores, comportamiento humano, relaciones y política). Los supuestos de conservación se centraron predominantemente en cuestiones a nivel de proyecto y de lugar, más que en las causas profundas de los problemas de conservación. Para los enfoques de conservación centrados en las personas y la equidad, nuestros hallazgos sugieren que los equipos de conservación deben comprometerse sistemáticamente con las áreas de mayor incertidumbre y centrarse especialmente en los supuestos axiomáticos sobre los contextos más amplios en los que operan los proyectos de conservación. Estas percepciones pueden servir de base para un diseño eficaz de los proyectos y para la adaptación a los cambios.

## INTRODUCTION

In this now‐or‐never decade of addressing global environmental challenges, conservation practitioners are feeling the push to achieve rapid progress toward conservation goals (Catalano et al., 2019; Chambers et al., 2022; Dickson et al., 2023). Although there have been noteworthy successes at regional and species‐specific levels (Bolam et al., 2021), global biodiversity and climate metrics continue to follow troubling trajectories (Allan et al., 2021; IPBES, 2019). The international community has set 2030 as a deadline to achieve a variety of biodiversity goals, including the United Nations’ 2030 Agenda for Sustainable Development targets and the goals established in the Convention on Biological Diversity's Kunming–Montreal Global Biodiversity Framework. Other actors, such as environmental nongovernmental organizations (eNGOs), are pursuing their own biodiversity goals in parallel with the international community and state actors and often work with a variety of local, state, and intergovernmental fora to develop and implement conservation projects around the world. For these actors to achieve these goals, progress must be realized not only in goal setting and consensus building but also in the successful execution of conservation projects on the ground.

Effective execution of conservation projects requires a thorough understanding of multiple human dimensions: the social and ecological contexts in which conservation projects exist, how change happens, the potential and limitations of proposed solutions, and what constitutes success (Dickson et al., 2023). Conservation projects invariably operate in complex and varying sociopolitical environments, so planning and implementation decisions take place against backdrops of uncertainty. Operating in such uncertain environments requires that conservation practitioners rely on multiple assumptions, though these assumptions are not always recognized or made explicit. They can also vary dramatically in theme and scope; some assumptions relate to the workings of the biological and ecological systems to be conserved, whereas others relate to the methodological assumptions about what is important to measure, what can be measured, and how best to measure it (Ban et al., 2017; Gallopín, 1996). Other assumptions are about people, their behavior, and how and why social change occurs (Albrecht, 1972; Solomon et al., 2015; Steg et al., 2014). And still others are preoccupied with implementation, including assumptions about where to deploy investments and to whom the costs and benefits of these projects should accrue (Law et al., 2018).

Given the importance of making rapid progress toward global biodiversity and climate goals, greater attention to the assumptions inherent in conservation action is called for. Some important literature has taken on this question, including work that explores the range of assumptions being made by practitioners in specific locales (Brias‐Guinart et al., 2022; Schröter et al., 2021) and those related to conservation in a particular domain (Armsworth, 2014; Waylen et al., 2013). Still, to our knowledge, no previous research has produced a large‐scale examination of conservation assumptions across multiple project types, conservation topics, and geographies.

To meet this need, we constructed a unique dataset of assumptions as identified by practitioners based at The Nature Conservancy (TNC) in 33 structured strategic planning workshops held from 2022 to 2024. As a global environmental nonprofit working in more than 80 countries, TNC's work involves a vast range of practices and topics and creates a unique opportunity to develop a broadly generalizable study of conservation assumptions across diverse projects and geographies. Using this dataset and a mix of grounded and structured qualitative methods, we sought to answer 3 questions: what types of assumptions do conservation practitioners identify in their work, what ontological patterns characterize conservation assumptions, and how do assumptions relate to project theories of change?

We considered our findings in the context of learning by doing and the importance of adopting an active and reflexive posture regarding the assumptions buried in conservation theories of change (Leisher et al., 2024). We also considered our findings in the context of the turn in recent years toward more people‐ and equity‐centered conservation (Corson et al., 2020; Moola & Roth, 2019; Shyamsundar et al., 2023).

## METHODS

**FIGURE 1:**
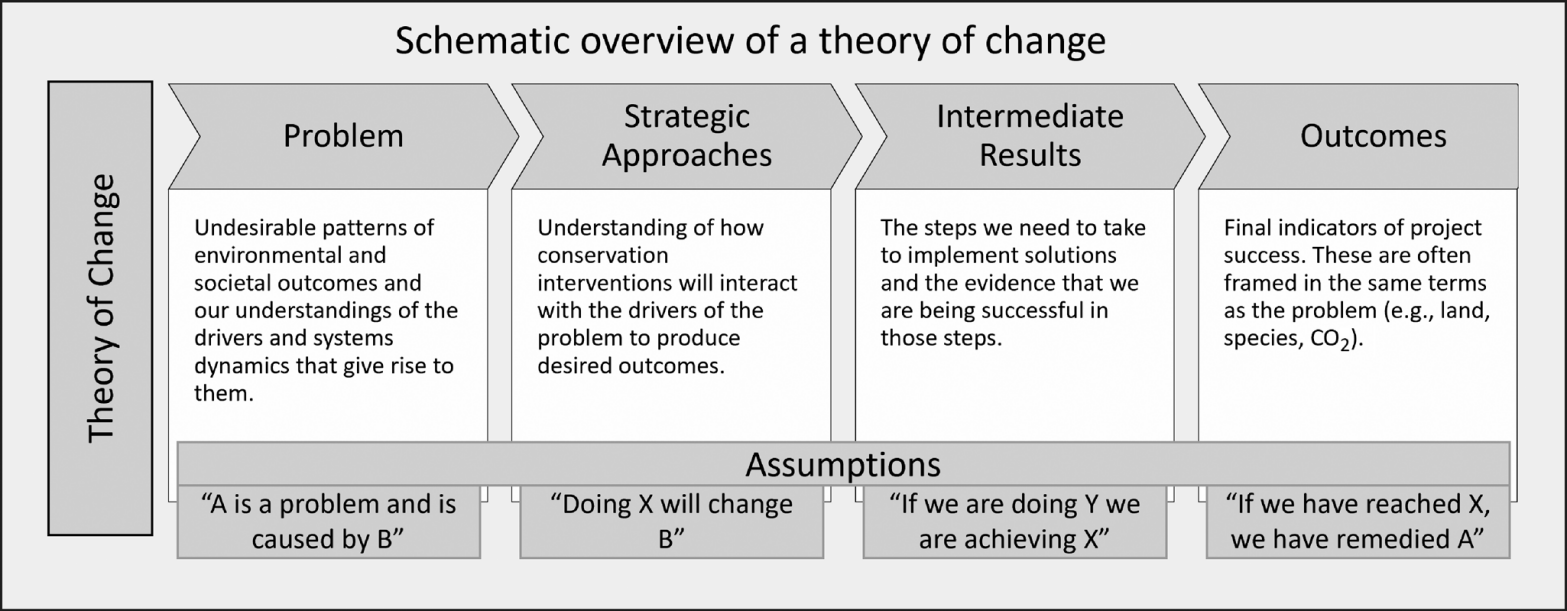
Theory of change framework based on Conservation Measures Partnership (2020) used in The Nature Conservancy's strategic project planning workshops. Details and examples from the dataset are in Appendix S1.

Our dataset contained the results of 33 TNC strategic project planning workshops conducted in English or Spanish or both from 2022 to 2024. These workshops are a common step in TNC's conservation planning process and are designed to enable conservation teams to reach consensus on key aspects of their projects and develop coherent strategies for how to maximize project success. Workshops are led by an expert facilitator, run for 2–3 days, and are attended by the project lead, project team members (often made up of a project manager, project administrator, and implementing team members), and TNC experts in adjacent or related teams, such as policy or finance. The facilitator guides group discussions with the goal of reaching consensus on the central problems the project seeks to solve and the key stakeholder group or groups the project must consider. Workshop groups also use TNC's theory of change framework (Figure 1) to develop and articulate a theory of change to serve as the foundation for their project's design. This discussion includes explicit description and articulation of the underlying assumptions embedded in the theory of change.

To identify assumptions, workshop facilitators asked participants “where are the hidden logic gaps in our theory of change?” Participants then review the theory of change they have constructed with the purpose of identifying unarticulated relationships among their problem statements, strategic approaches, intermediate results, and desired outcomes. The facilitator then guides the group to develop a full list of assumptions that should be made explicit in the team's theory of change.

The Nature Conservancy's theory of change framework (Figure 1) was developed by TNC scientists and staff based on an analysis of existing theory of change frameworks (Leisher et al., 2024). For the purposes of this research, we focused on 3 structural components of TNC's theory of change framework: strategic approaches, intermediate results, and outcomes. Strategic approaches refer to the key interventions employed to achieve the desired conservation impact. Intermediate results refer to the near‐term results a strategy hopes to achieve en route to its final objectives (Conservation Measures Partnership, 2020). Finally, outcomes refer to the measures by which a project or strategy will assess its final desired project impact. Assumptions can come into a plan in any (and likely all) of these stages and create dependencies that cascade throughout future stages. For example, an assumption about the root cause of a conservation problem will influence the strategic approaches preferred as well as the intermediate results that the team believes signal progress toward the desired outcomes. If the assumption proves incorrect, there may be a gap between the strategy chosen and the strategy necessary to fully achieve selected results and outcomes (Loken, 2023).

The workshop results were recorded by the workshop facilitator either on an online whiteboard template (i.e., a Miro board) or PowerPoint presentation. The 33 project teams span a diversity of global regions, scales (subnational to multicountry), and conservation project types (Table 1).

**TABLE 1:** Metadata for The Nature Conservancy teams participating in the 33 strategic planning workshops analyzed for conservation assumptions made in projects.

Conservation theme (number of workshops)	Region (number of workshops)	Jurisdictional setting (number of workshops)
Agriculture (3)	Africa (10)	Subnational (19)
Climate change adaptation (5)	Asia and Pacific Islands (including Australia) (7)	Country (3)
Climate change mitigation (4)	Central and South America (7)	Multicountry (11)
Energy (2)	North America (including Caribbean) (23)	
Equitable governance (5)		
Freshwater protection or management (14)		
Land protection or management (14)		
Marine and coastal protection or management (9)		
Sustainable development (5)		

*Note*: Numbers do not add up to 33 in some instances because workshop projects covered multiple themes or locations. All countries represented in workshops are as follows: Angola, Argentina, Australia, the Bahamas, Belize, Botswana, Brazil, Canada, Caribbean, Chile, China, Colombia, Ecuador, Gabon, Ghana, Guatemala, India, Indonesia, Kenya, Mexico, Micronesia, Mongolia, New Zealand, Paraguay, Peru, Seychelles, South Africa, Tanzania, the United States, and Zambia.

We constructed our dataset entirely from records kept during the strategic planning workshops. Because they were produced as a part of workshop participants’ regular work, they are the intellectual property of TNC and do not comprise human subjects data. Nevertheless, we submitted and received approval for our research design from TNC's Human Subjects Research team.

### Coding

We coded the 300 assumptions identified by conservation practitioners in the 33 workshops by assumption theme, assumption ontology, and their relation to TNC's theory of change framework. We used the MaxQDA platform (24.3.0) to conduct and organize our coding exercise. For each coding exercise, K.O. and B.P conducted a second round of coding to verify the results of the first round of coding. P.L. contributed a third round of code verification and hierarchical code categorization. Using MaxQDA's intercoder reliability tool, areas of coding disagreement were discussed and reconciled. Consensus was reached among all 3 authors for all coded materials. An overview of the research questions and their related methods is in Table 2.

**TABLE 2:** A summary of research questions related to the assumptions conservation practitioners at The Nature Conservancy (TNC) make in their projects and the analytical approaches or frameworks used to answer these questions.

Question	Approach or framework
What types of assumptions do conservation practitioners identify in their work?	Inductive analysis, thematic codes encountered in situ
What ontological patterns characterize conservation assumptions?	Deductive analysis, binary options: axiomatic or idiomatic
How do assumptions relate to project theories of change?	Mix of deductive and inductive analysis guided by TNC's theory of change framework

To examine assumption themes, we performed an inductive thematic analysis (Clarke & Braun, 2017). K.O. and B.P. started with an open coding approach to identify candidates for top‐level codes and then used MaxQDA's “suggest subcodes” feature to identify candidates for second‐level thematic categories. K.O. and B.P. then coded all assumptions into this structure, while continuing to identify emergent themes in vivo. Then, another round of code organization and consolidation was performed collaboratively by all authors.

To examine the ontological nature of the assumptions in our dataset, we used a binary coding categorization process. K.O. and B.P. designated each assumption as either, on balance, axiomatic (i.e., concerned with general principles) or idiomatic (i.e., concerned with local‐ or project‐level specifics) in nature. We used ratios to describe the results of our binary axiomatic–idiomatic coding effort because this expression enabled more direct and fair comparison across thematic categories with differing total assumptions.

To answer our question about how assumptions relate to components of project theories of change, K.O. and B.P. coded each assumption into the relevant component of TNC's theory of change framework.

### Positionality

The Nature Conservancy is a large eNGO headquartered in Arlington, Virginia (USA) and has a presence in over 80 countries and territories across 6 continents. The practitioners whose work generated the records that comprise our dataset are experts based at TNC, and we, the authors of this article, are employed by TNC. This work is therefore best understood as capturing the perspectives and assumptions made by agents of a large conservation‐focused eNGO. The range of assumptions that would be identified through similar exercises undertaken by smaller or regional eNGOs or by other types of actors (i.e., government) could be quite different. However, given TNC's global footprint of diverse conservation initiatives—which is reflected in this dataset—we believe it is possible to make some broader inferences regarding the range and prevalence of key themes.

Given TNC's origins and footprint, it is possible that our data may contain some bias toward U.S.‐based or Western conservation perspectives. This potential bias is likely reduced in part by TNC's organizational focus on understanding and integrating diverse viewpoints and interests into conservation strategy. The organization has developed and implemented a “Voice–Choice–Action” framework to elevate and integrate Indigenous perspectives into conservation; developed human rights assessment tools to allow practitioners to understand and assess human rights landscapes relevant to project footprints; and pursued equity and diversity initiatives in project considerations and internal planning. Together, these practices and tools help mitigate potential bias within our dataset.

Despite potential limitations the research below provides, at a minimum, a starting point for looking at the breadth of assumptions that conservation practitioners may be working with and potential gaps where assumptions are going unidentified or unquestioned.

## RESULTS

**FIGURE 2:**
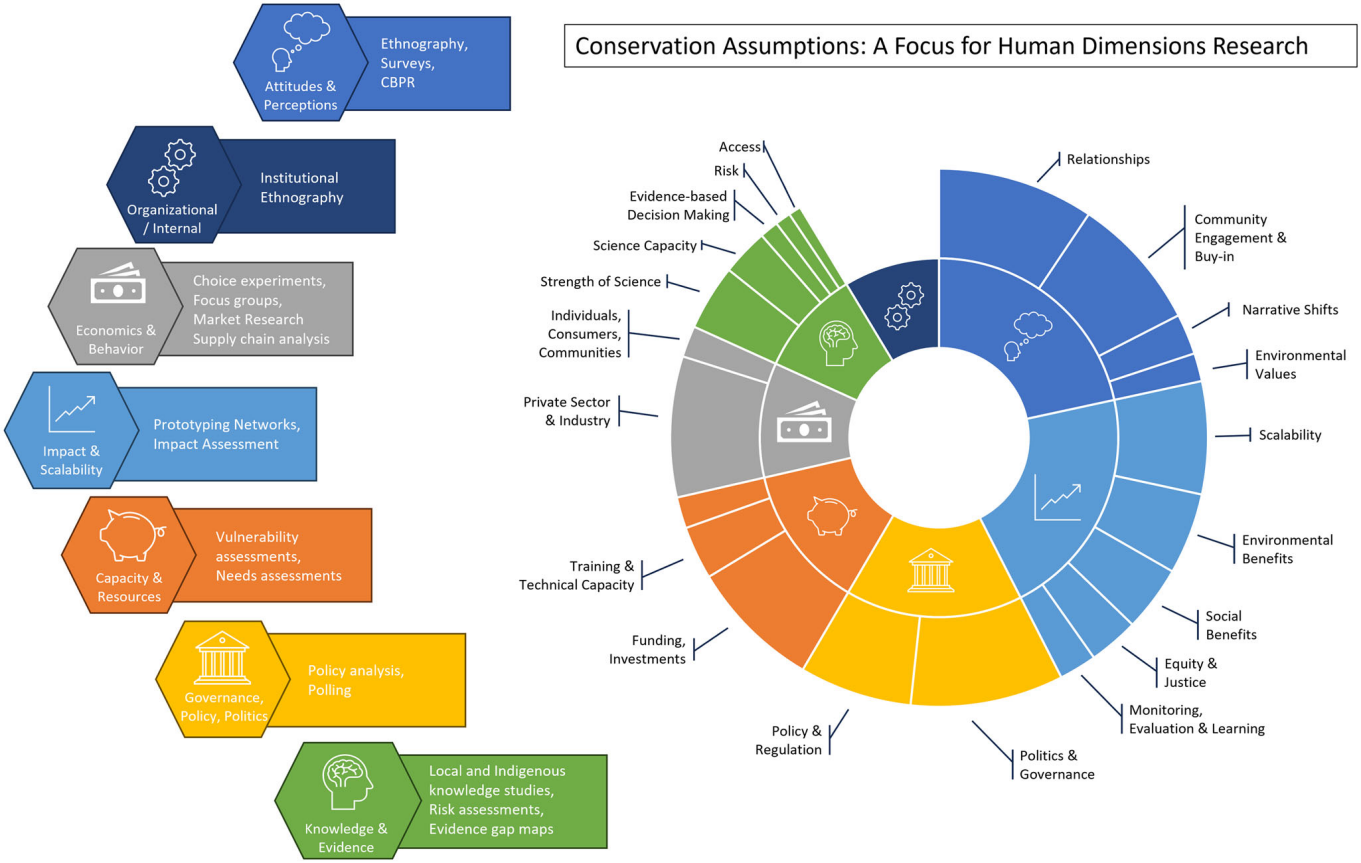
Thematic breakdown and relative prevalence of common conservation assumption themes indicated by conservation practitioners in strategic planning workshops conducted by The Nature Conservancy.

We identified 300 total assumptions across the 33 project workshops we analyzed. We organized these assumptions in 7 themes consisting of 20 subthemes in our inductive coding exercise (Figure 2; Table 4). The majority of the assumptions and assumption themes concerned the human dimensions of conservation, including 4 of the 5 most common assumption themes. These covered a wide variety of conservation‐related issues, including public attitudes and perceptions (the most identified assumption type, *n* = 106), impacts and scalability (*n* = 93), government, politics, and policy (*n* = 76), and economic and behavioral factors (*n* = 95).

We also identified a number of assumptions related to practical and administrative matters. The capacity and resources theme was the second most identified theme (*n* = 92) and pertained to human resource or technical skill capacities and funding availability for conservation projects. We also identified 46 organizational or internal assumptions made about how TNC structure and functioning could influence or impact project imperatives.

Though the majority of the assumptions and assumption themes we identified concern human dimensions, we also found 30 instances where practitioners identified assumptions about the knowledge and evidence being used in their projects, including 21 assumptions about the strength of the biological or physical science underlying their projects. Assumptions related to the strength of scientific understanding represent <10% of the total assumptions we identified.

Assumption totals exceed the number of assumptions in the dataset because some assumptions are coded into several thematic categories. Definitions of the 7 assumption themes we identified and examples from our dataset are in Table 3.

**TABLE 3:** Definitions and examples of themes covered in the conservation assumptions made by conservation practitioners at The Nature Conservancy.

Theme	Definition	Example
Attitudes and perceptions	Assumptions about the beliefs held by external (i.e., non‐TNC) stakeholders and rightsholders	“Media engagement will help positively shape narratives on carbon markets and create greater transparency.”
Impact and scalability	Assumptions about the impact projects will have and the extent to which these impacts can be scaled up	“What worked in X country will work in Y country, regarding adoption.”
Government, politics, and policy	Assumptions about the intersection of government and conservation projects	“[The federal agency] will move in lockstep with us on restoration and not be swayed by outside influences or shifting priorities.”
Capacity and resources	Assumptions about the extent to which projects can be supported with adequate human and financial resources	“Technological and economic investments will improve and can help us increase the pace and scale of change.”
Economics and behavioral factors	Assumptions about the economic interests of stakeholders and rightsholders and how those interests influence behavior	“Landowners will choose money over using their land in the way they always have.”
Knowledge and evidence	Assumptions about the quality, depth, and relevance of existing knowledge for project design	“Causes and magnitude of sedimentation are or can be correctly identified.”
Organizational or internal	Assumptions about how TNC's structure, norms, and rules may influence conservation projects	“TNC will advocate for policy based on researched and feasible solutions and will be able to effectively influence policy.”

### Ontological patterns

We identified noteworthy ontological differences in the assumptions in our dataset. Specifically, our results showed that practitioners identified idiomatic assumptions more than axiomatic assumptions by a factor of more than 4:1 across the dataset. This ratio varied by thematic category, ranging from a low of 2.1:1 (for assumptions related to impacts and scalability) to 22.5:1 (assumptions related to organizational or internal factors [Table 4]). Idiomatic assumptions were more common than axiomatic assumptions for each thematic category of assumptions.

**TABLE 4:** Results of the coding exercise seeking to identify and describe the conservation assumptions made by conservation practitioners at The Nature Conservancy.

	Research question 1	Research question 2			Research question 3				
Assumption theme	Frequency	Axiomatic (A)	Idiomatic (I)	I:A ratio	Enabling conditions	Strategic phase	Intermediate phase	Outcomes	Broader impacts
Public attitudes and perceptions	106	23	91	4.0:1	47	56	23	15	12
Environmental values	9	3	6	2.0:1	6	7	0	2	2
Narrative shifts	13	5	10	2.0:1	6	7	3	2	1
Community engagement and buy‐in	43	10	38	3.8:1	18	28	10	4	4
Relationships, trust, and alignment	50	5	46	9.2:1	20	28	13	3	7
Capacity and resources	92	9	88	9.8:1	45	48	27	8	10
Training and technical capacity	17	2	16	8.0:1	7	8	8	1	3
Staff and other human resources	10	0	10	–	5	9	1	0	0
Funding, investments, and other capital	42	5	41	8.2:1	18	15	14	6	6
Economic and behavioral factors	55	19	47	2.5:1	15	28	13	9	11
Consumer, community, and individual behavior	10	6	7	1.2:1	4	8	1	1	0
Private sector or industry incentives	45	13	40	3.1:1	11	20	12	8	11
Governance, politics, and policy	76	12	67	5.6:1	31	40	20	11	13
Politics and governance	46	8	41	5.1:1	25	24	13	4	6
Policy and regulation	34	4	30	7.5:1	8	19	8	7	7
Impacts and scalability	93	34	73	2.1:1	10	32	21	39	26
Equity and justice impacts	17	4	11	2.8:1	4	12	1	3	0
Environmental benefits	26	16	18	1.1:1	0	11	1	13	8
Monitoring evaluation and learning	12	2	10	5.0:1	5	3	6	2	1
Social benefits	21	11	14	1.3:1	1	8	3	9	4
Knowledge and evidence	30	10	24	2.4:1	3	15	4	9	4
Strength of science	21	8	16	2.0:1	2	13	3	5	2
Risk	5	0	5	–	1	1	1	2	1
Decisions based on correct evidence	6	4	4	1:1	0	3	0	3	1
Knowledge generation capacity	5	1	4	4.0:1	2	3	2	0	0
Knowledge and evidence availability	14	5	11	2.2:1	0	8	1	4	2
Organizational or internal	46	2	45	22.5:1	23	26	9	4	1
All assumptions	300	64	260	4.1:1	109	149	67	54	45

*Note*: Dataset contains 300 assumptions, but each column does not necessarily add up to 300 because each assumption can receive multiple codes. Research questions are described in the INTRODUCTION section and listed in Table 2.

### Assumptions by theory of change component

With respect to how these assumptions overlapped with the different structural components of a theory of change (Figure 1), assumptions tied to strategic approaches were most prevalent (*n* = 149), followed by assumptions about intermediate results (*n* = 67). Assumptions related to project outcomes were least common (*n* = 54). We found no evidence, however, that specific assumption themes were more or less prevalent for different stages of TNC's theory of change framework.

We encountered 2 categories of assumptions that that did not clearly fit the a priori theory of change framework utilized in TNC planning. The first of these types we called “enabling conditions for project success” (*n* = 109) (hereafter enabling conditions); these pertained to factors beyond the project sphere of influence but that were crucial to enabling (or precluding) project success, such as cultural narratives, political will, and geopolitics. The second group, which is a subset of the first, we called “enabling conditions for broader impact” (*n* = 45) (hereafter, broader impacts); these are assumptions about whether project outcomes will be viable at scale and have sustained impact after the end of the specific intervention. Table 4 contains a full summary of results, and Appendix S1 has extended definitions and descriptions of the enabling conditions and broader impact categories.

Finally, we found no statistically significant differences in the prevalence of thematic or ontological assumptions in different geographic regions or for different kinds of conservation projects (i.e., protection, restoration, policy). This finding suggests that the thematic patterns of assumptions, as well as relative prominence of idiomatic assumptions relative to axiomatic ones, are common across the cases in our dataset

## DISCUSSION

The impacts of human activities on climate and biodiversity have already reached magnitudes that were unthinkable a few decades ago, and time is short to reverse these trends. To maximize the chance of project success, it is critical that conservation practitioners design monitoring, evaluation, and learning (MEL) practices into conservation projects to enable learning and inform strategic adjustments through a project's lifespans (Dickson et al., 2023; Rice et al., 2020). And, critical within these MEL practices is attention to the assumptions that are central to a project's theory of change. As Dickson et al. (2023) point out, faulty assumptions can infiltrate and disrupt all aspects of a project, including the veracity of the scientific basis of the intervention, the project resources being deployed, and the impact or permanence of outcomes. To our knowledge, our work is the first to offer a detailed examination of the patterns of assumptions being made across a series of conservation projects, the extent to which those assumptions are project specific or relate to more theoretical or generalizable claims about people and society, and how assumptions relate to how change happens.

The vast majority of assumptions identified by workshop participants are related to what has been called the human dimensions of conservation (Huntington et al., 2007). Although others have provided essential guidance on social science and how to bridge the interdisciplinary differences involved in bringing social sciences to conservation (Bennett et al., 2017; Moon & Blackman, 2014), there remains a need for practical guidance on how and where to apply these highly diverse methodologies and methods in our work. Our research can help address this gap because it provides a comprehensive typology of how and where in conservation work human dimension issues manifest (Figure 2). With this new understanding, we believe that conservation practice can be elevated in multiple ways. First, teams can be more systematic and exhaustive in anticipating potential areas of uncertainty in their strategic plans and theories of change and then can seek out appropriate bodies of existing social science knowledge to minimize uncertainty or revise those assumptions. For example, if a project rests on an assumption that the root cause of unwanted behavior is economic in nature and operating at the level of individual actors (e.g., farmers), that assumption could be evaluated in light of additional established social science pointing to the potential role of structural factors, political ecology, and public discourse.

Second, the typology of assumptions we present has a practical advantage in that it can help practitioners who may not be aware of the broad range of social sciences that may be of use for ensuring that MEL efforts track whether a project's identified assumptions are holding true. For example, when working with assumptions about people's attitudes and perceptions, ethnographic and community‐based participatory methodologies would likely be appropriate. Assumptions about knowledge and evidence could be checked through the methods of science and technology studies and by bringing in Indigenous or local ecological expertise. Likewise, attending to assumptions about capacity and resource availability could involve methods for needs assessment, sustainable livelihoods assessments, or vulnerability assessments.

What we did not find, or only found rarely, in our data also offers insights. For example, assumptions about the strength of natural and physical science knowledge occurred infrequently. We suggest that this should be an area of concern, in that teams should remain duly skeptical of the strength and place‐based appropriateness of the natural and physical science basis of the interventions they pursue. Science and data are neither impartial or apolitical (Loring et al., 2021; Moore et al., 2011). Likewise, there are no panaceas for conservation challenges, and the science behind many contemporary climate and biodiversity solutions, such as regenerative farming, reforestation, and marine protected areas, is contested and unsettled (Hasler et al., 2024; Rehberger et al., 2023; Relano & Pauly, 2023). Although local‐scale conservation projects have to rely on the best available science at the time of their inception, we argue that active skepticism and attention to unrecognized neoliberal biases (Moore et al., 2011) and phenomena like negative learning (Oppenheimer et al., 2008) should become a priority for conservation organizations, especially those with the capacity to incorporate basic research around these matters into their project design. This is an area where the social sciences, specifically the field of science and technology studies, have much to offer in terms of concepts and methods for being more reflexive about and attentive to what even the best available science can and cannot contribute when it comes to planning and prioritization.

It is now widely recognized that for conservation to be effective and sustainable, our projects need to be closely attuned to local social, cultural, and political contexts and histories (Dawson et al., 2021; Parks & Tsioumani, 2023). We believe this is reflected in our data, which contains two additional categories of assumptions not directly tied to TNC's adopted structure for developing theories of change: assumptions for enabling conditions and assumptions regarding the broader impacts of conservation projects. In these two categories, we see a wide range of awareness of diverse issues that, although often outside the scope of the conservation intervention itself, are still relevant to project success, including market forces, issue salience, and politics at multiple scales.

**FIGURE 3:**
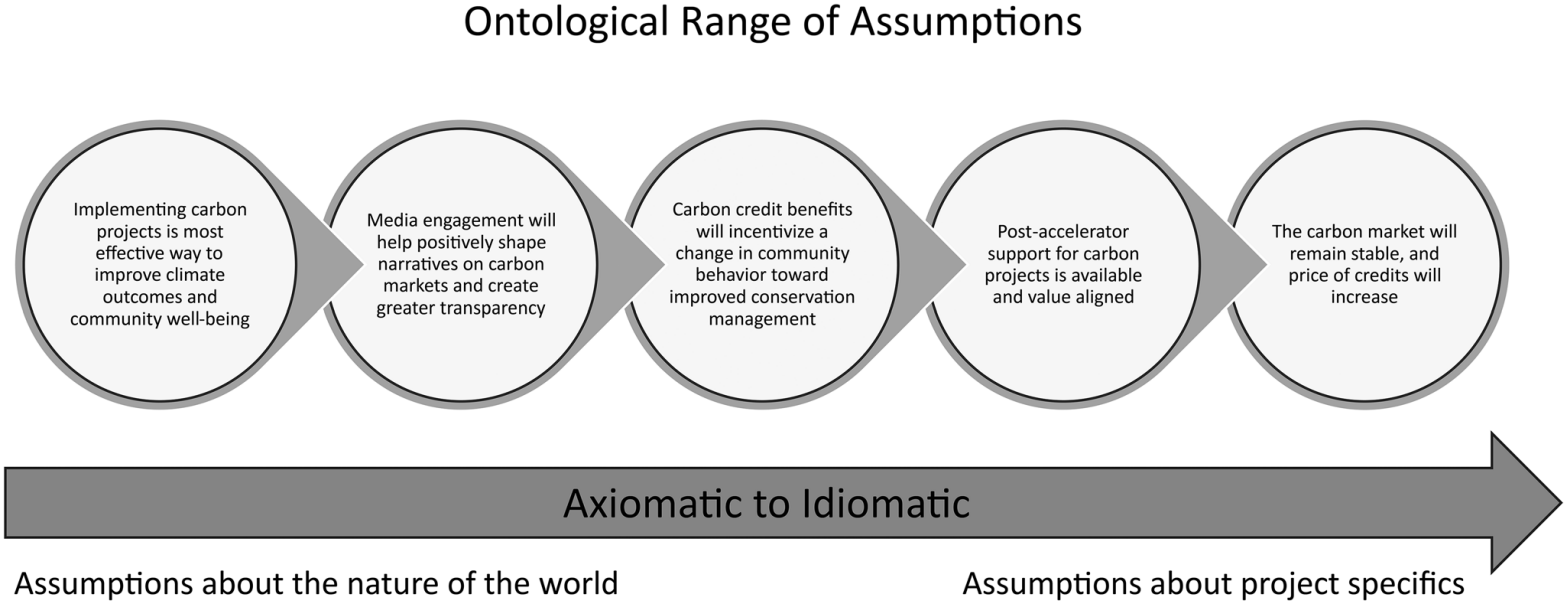
Nesting of conservation assumptions in ontological chains that are linked topically but sequenced ontologically. On the left side, axiomatic assumptions relate to enabling conditions and broad assumptions about the function and effectiveness of a specific solution. On the right side, assumptions become more specific to system behavior and responses specific to the intervention. The chain of assumptions pictured are from a project related to carbon markets.

And yet, our data also showed that practitioners frequently focused on idiomatic, or site‐ and project‐specific assumptions, rather than the deeper axiomatic assumptions they may be making about how their project relates to the context in which it operates (see Figure 3). This finding aligns with those of Bennett et al. (2022), who in a review of human dimensions research being pursued by social scientists affiliated with the Smithsonian Working Land and Seascapes Initiative observed that more attention was paid by researchers to project‐specific research questions, whereas much less attention was paid to what they call “curiosity‐driven” research that can produce generalizable insights about the role of people in conservation.

If practitioners are far more often focusing on assumptions concerned with project specifics rather than assumptions about the role of systems and power, for example, there is a risk that the root causes of environmental problems will go misunderstood or overlooked. At a minimum, this surficial perspective could create a strategy gap between the strategies adopted to address a conservation problem and those necessary to actually solve it (Loken, 2023). For example, a project might identify the assumption that a particular pricing for a payment for ecosystem services would be sufficient to alter farmer behavior; however, the deeper assumption that pricing reflects the dominant driver in their current decision‐making could be wrong. Research with farmers, for example, shows that multiple such issues come into a farmers’ thinking when deciding whether to adopt new practices, from concerns about retirement, cultural values about what constitutes so‐called good farming, and trust in the messengers promoting the new practices (Drescher et al., 2024; Li & Wang, 2024; Miller‐Klugesherz & Sanderson, 2023). Research that focuses only on the monetary aspect of this decision—what level of payments would be necessary to provoke adoption—will necessarily miss these deeper and potentially more important drivers, possibly leading to the determination that farmers are simply not interested in the practice or conservation to begin with.

These findings in particular offer essential guidance for teams wishing to think deeply about the assumptions they identify and to anticipate the kinds of social science partners who could push their thinking and help minimize or otherwise redress these assumptions from project to project and place to place. If teams are accurately identifying superficial assumptions but missing their deeper underpinnings, their ability to monitor, evaluate, and learn from how those assumptions play out over the course of the work is limited and risks to success can accumulate unnoticed. Thus, the potential upside from investment and focus on greater use of social science is significant because a more robust ability to identify and understand the diverse social and political factors complicating conservation projects can ideally shape project strategy and design and improve project success.

As a field, conservation has long stressed the importance of working with the best available science (Charnley et al., 2017; Sutherland et al., 2004); likewise, approaches such as adaptive management and theories of change frameworks, are increasingly prevalent in conversations about how to promote rapid learning from both conservation successes and failures (Rice et al., 2020; Wardropper et al., 2022). Insights from the social sciences are key to unlocking deeper learning opportunities—that is, to identify and check the assumptions, both explicit and implicit, that stitch together the causal logic of conservation and climate interventions (Pahl‐Wostl, 2009).

Always looking deeper with one's research can ensure that one does not get stuck in a mode of learning known as single loop learning. The multiloop model of learning (Pahl‐Wostl, 2009) offers a model for differentiating how to approach learning from outcomes in a system—in this case, the success or failure of a conservation assumption or entire project. In the simplest sense, triple loop learning identifies 3 levels of learning: what is being done (first level), how it is being done (second level), and why it is being done (third and deepest level). Single‐level learning—revisions to what is being done that do not involve revisiting the assumptions that underpin the current approach—is the most common kind of learning, as is reflected in our data. Double loop learning, by comparison, which requires thinking past idiomatic assumptions and reevaluating the axiomatic underpinnings of the chosen strategy, is present but less common.

In recent years, the conservation field as a whole has also been engaged in the third and deepest kind of learning—triple loop learning (or learning for transformation)—which moves from the how of one's strategies to the why. The turn toward human dimensions, equitable conservation, and inclusion of Indigenous Peoples and local communities is arguably an example of deep reflection on the ethics and operating principles of the conservation community (Corson et al., 2020; Moola & Roth, 2019; Shyamsundar et al., 2023). What our research suggests is that this deeper learning and evolution within the field has yet to kick off new learning in the second loop, in which practitioners actively revisit the appropriateness of current approaches in light of these new operating principles. More learning of this kind is critical because no matter how committed organizations are to reforming the ethical foundations of their work, if conservation practitioners do not identify and address the deep biases that prefigure their understandings of conservation problems and how they ought to be solved, neither the ethical reinvention nor the practical outcomes sought will be achieved.

Our findings represent an important advance in the understanding of the assumptions that underpin conservation projects at large eNGOs, which are a crucial actor for biodiversity progress. Further, our findings offer both practical and theoretical insights that can empower conservation practitioners to develop implementation plans that are self‐aware of the assumptions they rely on and proactive about learning as these assumptions are tested in practice. Together, this work can enable critique and close examination of the upcoming conservation initiatives that will determine the extent to which major biodiversity goals are reached.

## Supporting information



Supplementary Materials
