# Supplementary material for: Examining critical assumptions in global conservation practice
Source: Conserv Biol. 2025 Jul 3;39(6):e70102. doi: 10.1111/cobi.70102 (PMC12658941; doi:10.1111/cobi.70102)
Supplement: Supplementary file 1 — Supplementary Materials [file COBI-39-e70102-s001.docx]

**Supplementary information**

**Table SI-1.** Definitions and examples of the structural components of a theory of change

| **Assumption Class** | **Description** | **Examples** |
| --- | --- | --- |
| Strategic assumption (n=149) | Relating to causal claims about problems and interventions | - Carbon credit benefits will incentivize a change in community behavior toward improved conservation management - Forest restoration may require the creation of new private sector entities that currently don't exist and new models of public/private partnership - Inclusive planning that incorporates local knowledge and priorities will reduce conflicts and negative impacts and lead to enduring conservation outcomes |
| Intermediate assumptions (n=67) | Relating to activity- and progress-related indicators that can be monitored during a project to support adjustments and adaptation | - Implementers will have continued interest in carbon projects - We can measure [climate] resilience and how it changes - We will establish trusting relationships with community members via communication |
| Outcome assumptions (n=54) | Assumptions related to the project’s outcomes and whether they are effective at contributing to broader goals | - The carbon market will remain stable [after the end of the project] and price of credits will increase - Local communities will benefit from aquaculture development - We correctly prioritized landscapes to achieve global scaling |
| Enabling condition assumptions (n=109) | Assumptions related to the social and ecological circumstances that will support or accelerate project success | - Conditions are in place to support vibrant forest markets - [Our project] will resonate with political ambitions - Communities agree to work with us and one another |
| Broader impact assumptions (n=45) | Assumptions related to the scalability and sustainability of solutions and their knock-on benefits. A sub-type of enabling condition assumptions | - Externalities (like hydropower infrastructure, mining, etc.) that impact freshwater fisheries can be effectively managed - Trees planted are resilient in 50+yrs - Long term funding can be secured for enforcement and education |
